# Lipopolysaccharide Is Cleared from the Circulation by Hepatocytes via the Low Density Lipoprotein Receptor

**DOI:** 10.1371/journal.pone.0155030

**Published:** 2016-05-12

**Authors:** Elena Topchiy, Mihai Cirstea, HyeJin Julia Kong, John H. Boyd, Yingjin Wang, James A. Russell, Keith R. Walley

**Affiliations:** Centre for Heart Lung Innovation, St. Paul’s Hospital, University of British Columbia, Vancouver, Canada; Innsbruck Medical University, AUSTRIA

## Abstract

Sepsis is the leading cause of death in critically ill patients. While decreased Proprotein Convertase Subtilisin/Kexin type 9 (PCSK9) function improves clinical outcomes in murine and human sepsis, the mechanisms involved have not been fully elucidated. We tested the hypothesis that lipopolysaccharide (LPS), the major Gram-negative bacteria endotoxin, is cleared from the circulation by hepatocyte Low Density Lipoprotein Receptors (LDLR)—receptors downregulated by PCSK9. We directly visualized LPS uptake and found that LPS is rapidly taken up by hepatocytes into the cell periphery. Over the course of 4 hours LPS is transported towards the cell center. We next found that clearance of injected LPS from the blood was reduced substantially in *Ldlr* knockout *(Ldlr-/-)* mice compared to wild type controls and, simultaneously, hepatic uptake of LPS was also reduced in *Ldlr-/-* mice. Specifically examining the role of hepatocytes, we further found that primary hepatocytes isolated from *Ldlr-/-* mice had greatly decreased LPS uptake. In the HepG2 immortalized human hepatocyte cell line, LDLR silencing similarly resulted in decreased LPS uptake. PCSK9 treatment reduces LDLR density on hepatocytes and, therefore, was another independent strategy to test our hypothesis. Incubation with PCSK9 reduced LPS uptake by hepatocytes. Taken together, these findings demonstrate that hepatocytes clear LPS from the circulation via the LDLR and PCSK9 regulates LPS clearance from the circulation during sepsis by downregulation of hepatic LDLR.

## Introduction

Septic shock is a complication of a severe microbial infection (sepsis) that triggers an uncontrolled systemic inflammatory response and subsequent organ failure. Effective treatment of severe sepsis can lead to complete resolution while ineffective treatment can be fatal [1]. However, beyond antibiotic therapy there are currently no effective treatments for septic shock. To prevent severe inflammation during infection, the patient must quickly clear bacterial endotoxins from their circulation before they accumulate and are able to interact with immune cells and vascular endothelium and induce inflammatory organ failure. Bacterial endotoxins, such as lipopolysaccharide (LPS), are carried within lipoprotein particles [2]. Thus, one mechanism of action for sepsis treatments could be acceleration of lipoprotein clearance by hepatocytes. Recently it has been shown that decreased function of proprotein convertase subtilisin/kexin type 9 (PCSK9) increases survival of patients with septic shock [3]. Pharmacological inhibition of PCSK9 improves survival and inflammation in a murine polymicrobial peritonitis model, while *Pcsk9* knockout mice display decreased production of inflammatory cytokines in response to LPS (3). PCSK9 binds the Low Density Lipoprotein Receptor (LDLR) and promotes LDLR lysosomal degradation [4] so decreased PCSK9 function increases hepatocyte LDLR density [5]. We and others [3, 6, 7] have hypothesized that one possible mechanism of the beneficial effect of decreased PCSK9 function in sepsis is increased clearance of pathogen lipids, such as LPS, via the LDLR on hepatocytes. Indirect evidence is supportive, however, no studies have directly examined hepatic uptake of LPS via the LDLR so this hypothesis remains an intriguing conjecture.

LPS is carried in the blood within lipoprotein particles including LDL [6]. The liver plays a role in LPS clearance [8–10] and the primary target of PCSK9 is the LDLR [4]. Therefore it is reasonable to speculate that LPS carried in LDL particles is cleared by hepatocytes via the LDLR [5, 7, 11]. Indirect evidence includes the observation that the beneficial effect PCSK9 Loss-of-Function genetic variants in septic humans is not observed in patients who also carry a specific mutation of the LDLR receptor (rs688) which impairs PCSK9 binding–thus implicating a role for the LDLR [3]. The number of patients carrying both specific PCSK9 and LDLR genetic variants was not large so that this indirect inference is not certain. Further indirect evidence supporting this hypothesis includes the observation that PCSK9 inhibition abolishes the effect of *Ldlr* gene knockout on the physiologic effects of LPS administration in mice [3]. None of these observations are specific for hepatic clearance of LPS. Inference of a role for the LDLR from PCSK9 studies is further uncertain because PCSK9 also binds the Very Low Density Lipoprotein Receptor (VLDLR), ApoE Receptor 2, and others [6]. Furthermore, LPS and other pathogen lipids are carried in HDL and VLDL, in addition to LDL [7]. Thus, it is unknown whether previous observations are accounted for by LPS clearance by hepatocytes via the LDLR. Accordingly, we tested this hypothesis directly, proposing an alternative mechanism of initial uptake and clearance of LPS into hepatocytes.

## Materials and methods

### Animal model

All animal studies were approved by the University of British Columbia animal ethics committee. Male *Ldlr* knock-out (*Ldlr*-/-;B6;129S7-*Ldlr*^tm1Her^/J) and genetic background control mice, body weight 25–30 grams, 10–16 weeks old, were purchased from Jackson Labs (Bar Harbor, ME).

### In vivo hepatic LPS uptake

Mice were anesthetized and kept warm using a heating pad. 5 mg/kg FITC-conjugated *E*.*coli* LPS (Sigma, F8666) or non-fluorescent LPS (Sigma L2880) for the control group were injected into a tail vein. Liver and blood were harvested at 1 hour and 6 hours after LPS injection. Liver tissue was frozen in liquid nitrogen and, at the time of assay, 30–40 mg tissues pieces were homogenized in 0.5 mL PBS. Blood was collected into heparinized tubes. Plasma was separated by centrifugation for 10 minutes at 2,000 g using a refrigerated centrifuge at 4°C. Fluorescence was detected using a Tecan GENios microplate reader with a flat bottom black 96 well assay plate (Costar 3916).

### Western Blot analysis

Frozen liver tissue, 20–25 mg, was homogenized in 500 μL RIPA buffer supplemented with protease inhibitor cocktail (Sigma), centrifuged at 14,000 rpm for 10 min at 4°C and supernatant collected. For Western blot analysis equal protein amounts were separated by SDS-PAGE and transferred onto a membrane. The membrane was blocked for 1 hour in phosphate-buffered saline PBS-T (0.1%) with 5% milk, followed by immunostaining with optimized dilutions of murine VLDLR primary antibody (Origene TA309928) in 5% milk in PBS-T overnight at 4°C. Then the membrane was incubated with a secondary antibody (Abcam, ab97265) in 5% milk in PBS-T for 1 hour at room temperature.

### LPS uptake by HepG2 cells

HepG2 immortalized human hepatocytes (American Type Culture Collection HB-8065) were grown to confluence on 24-well plates in DMEM medium (Invitrogen, 11965–065) supplemented with 10% FBS. Then the media was removed and replaced with 80% DMEM and 20% human plasma from healthy donors. Cells were then treated with 2.5 μg/mL ultrapure Alexa-488 Fluor^™^ LPS (*Escherichia coli* strain from Life Technologies L23351) or with non-fluorescent LPS as a control for 24 hours total. Cellular LPS uptake was assayed via flow cytometry. Cells were gated by forward and side scatter for viability using previously determined parameters; 10,000 gated cells were counted per sample. The output of interest was median fluorescence intensity (MFI) from the instrument’s FL1 laser (excitation wavelength, 488 nm, emission wavelength, 525/20 nm). Background autofluorescence of cells treated with nonfluorescent LPS was subtracted to determine the fluorescence level resulting from uptake of the LPS conjugate. Data analysis was performed using Kaluza Analysis 1.3 software (Beckman Coulter). In separate experiments, 24 hours after siRNA transfection cells were treated with 3 μg/mL purified recombinant human PCSK9 protein (ACROBiosystems PC9-H5223) as described previously [12].

### LPS-LDL uptake by HepG2 cells

1mg of LDL isolated from freshly obtained plasma from healthy donors was labeled with 10ug Alexa-488 Fluor LPS (*Salmonella* Minnesota from Life Technologies L23356) at 37°C overnight. After the incubation, the mixture was passed through the G-75 Sephadex column for separation of lipoprotein-bound LPS and unbound LPS. The G-75 Sephadex beads were expanded in 150 mM NaCl and 1 mM EDTA at pH 7.4 and were added to 5 mL serological pipet up to 5 mL. The column was washed with 150 mM NaCl and 1 mM EDTA at pH 7.4. Twelve fractions were collected; protein concentration and fluorescence of each fraction were measured by BCA assay and microplate reader with a flat bottom black 96 well assay plate (Costar 3916) respectively. The fraction which exhibited overlapped peaks of protein concentration and fluorescence level was chosen for further experiments (See S1 Fig). HepG2 cells were plated in 24-well plates at 200,000 cells/well density. The cells were grown for 2 days to full confluence. At 100% confluence, cells were washed twice wish PBS and 3 μg/mL PCSK9 or 3 μg/mL LDLR (R&D systems BAF2148) antibody was added for 2 hours. After 2-hour-pretreatment, the cells were treated with 50 μg/mL LDL-LPS for 24 hours. After a 24-hour-incubation, the cells were washed twice with cold PBS and then used for flow cytometry analysis.

### siRNA reverse transfection

LDLR Trilencer-27 Human siRNA was obtained from Origene (ID 3949). Silencer-selected negative control siRNA was purchased from Ambion (catalog no. 4390843). HepG2 cells were plated at 30–40% density together with siRNAa/Lipofectamine RNAiMAX mix (Life Technologies) at a final siRNA concentration of 6 nM. Gene knockdown was confirmed using flow cytometry (9Beckman Coulter Gallios Flow Cytometer) after 48–72 hours using an LDLR-PE conjugated rabbit anti-human antibody (SinoBiologicals 10231-R3031-P).

### Primary hepatocytes isolation and culturing

Hepatocytes were isolated from mice by *in situ* collagenase perfusion technique (collagenase Type I, Life Technologies 17100–017), modified as described previously [13]. Hepatocytes (150,000 cells/mL) were plated on 12 mm coverslips precoated with Collagen I (Neuvitro H-12 Collagen) in DMEM medium with 20% Bovine Serum, 100 units/mL penicillin and 100 units/mL streptomycin. Cells were allowed to attach to coverslips for 6–12 hours prior to treatment.

### LPS uptake by primary hepatocytes

Primary hepatocytes were treated in the same manner as HepG2 cells with purified recombinant human PCSK9 protein (3 μg/mL) for 3 hours, then 2.5 μg/mL Alexa Fluor 488–conjugated LPS was added for 1, 6 and 24 hours total. Coverslips were washed twice with PBS, fixed with 4% paraformaldehyde for 20 minutes and stained with DAPI stain for 30 seconds. LPS uptake was visualized using a Leica inverted fluorescence microscope. As an additional control, cells were treated with non-fluorescent LPS in the same manner. No fluorescence was detected and images were used as control blanks. The investigator was blinded to treatment groups when performing imaging. Images were analyzed using VOLOCITY software (PerkinElmer Inc.). For the live cell-imaging experiments primary hepatocytes were plated on 35 mm glass bottom dishes pre-coated with collagen I (MatTek). Nuclei were stained with Hoescht stain for 30 seconds prior to addition of Alexa Fluor 488–conjugated LPS (2.5 μg/mL to 4 μg/mL to increase the signal). LPS uptake was visualized every 15–30 minutes for 6 hours and images analyzed as above.

### Statistical analysis

Significant differences between control and treatment groups were assessed using a two-tailed Student's t-test. Data from multiple-group experiments were analyzed using one-way ANOVA, followed by a post hoc Tukey test to compare groups. P < 0.05 was considered statistically significant.

Supplemental Material and Methods section is available online.

## Results

### Time course of hepatocyte uptake of LPS

**Fig 1:**
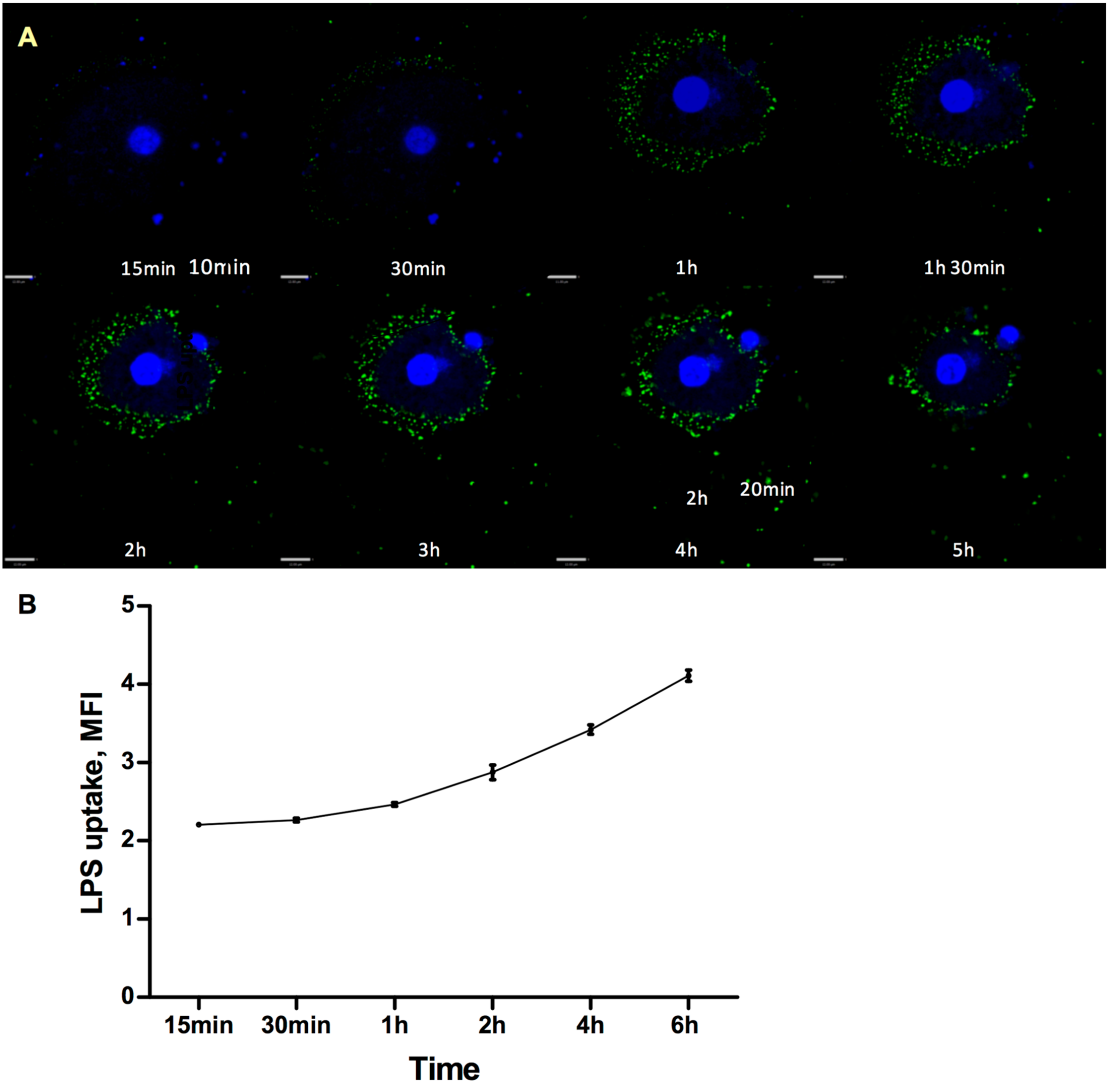
Time course of LPS uptake by hepatocytes. (A) For a live-imaging experiment fresh isolated primary hepatocytes were plated on 35 mm glass bottom dish precoated with collagen I (MatTek) and incubated overnight. The cells were stained with Hoechst to identify nuclei (blue) prior Alexa Fluor 488-conjugated E.coli LPS (green) addition and then immediately imaged constantly with the 63x objective using a Leica Inverted Fluorescence microscope, at 37°C and 5% CO2. (B) Time course of LPS uptake by HepG2 cells. HepG2 cells were treated with Alexa-488 FluorTM E.coli LPS (2.5 μg/ml) for 15, 30 min, 1, 2, 4 and 6 hours in media containing 20% human plasma. LPS uptake was measured by flow cytometry. Data presented as mean fluorescence intensity, mean±SEM (n = 3).

To test our hypothesis we first performed live-cell confocal imaging of freshly isolated murine primary hepatocytes treated with Alexa488-conjugated LPS *ex vivo*. LPS internalization could be detected as early as 30 minutes using confocal microscopy and continued to increase during the 6 hours following LPS administration (Fig 1A). We then measured the Alexa488-conjugated LPS uptake by immortalized human liver HepG2 cell line using flow cytometry (Fig 1B). Intracellular LPS levels gradually increased in a time-dependent manner over the course of the experiment.

### Murine Ldlr knockout (*Ldlr-/-*) reduces plasma clearance of LPS

**Fig 2:**
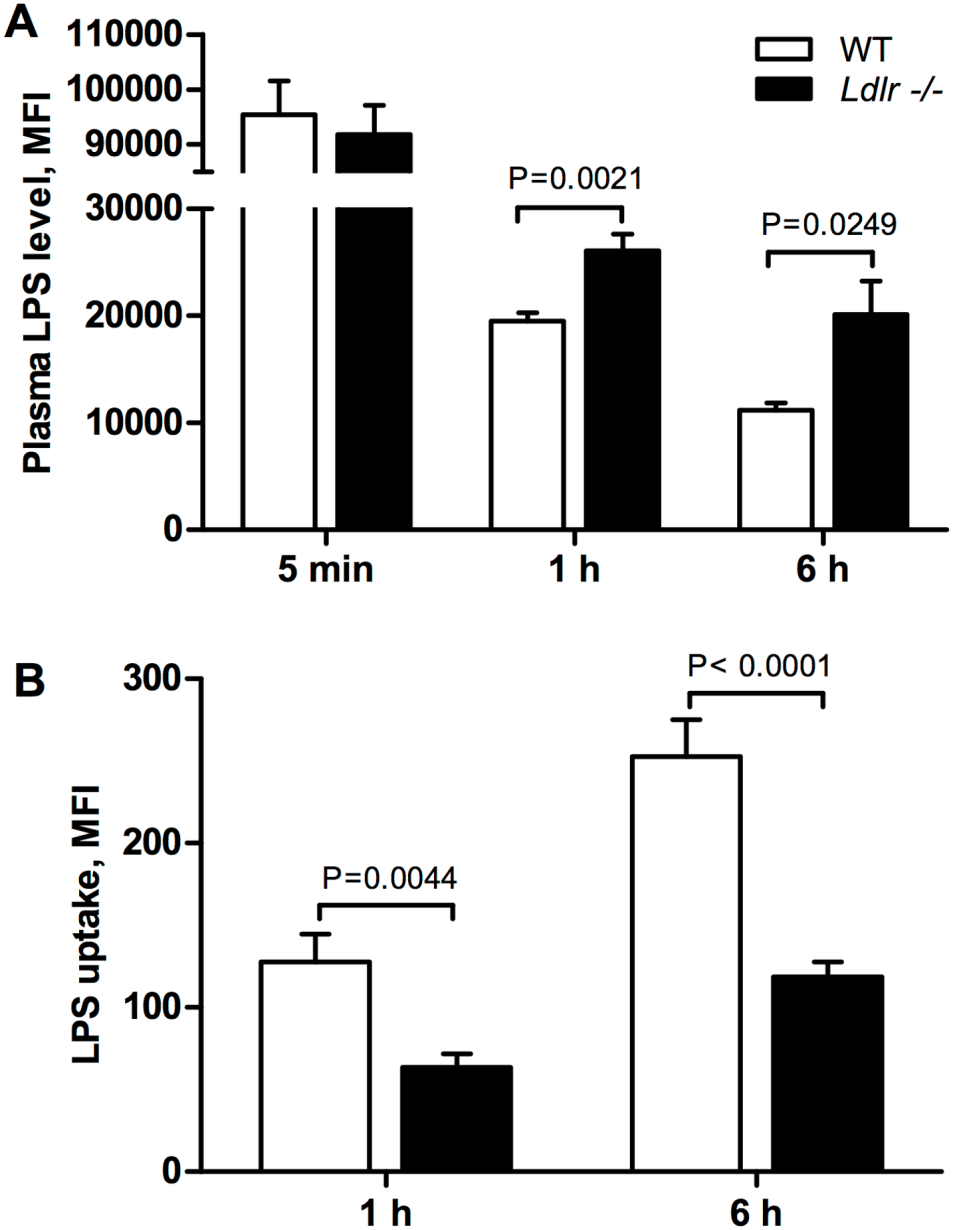
Ldlr knockout (Ldlr-/-) reduces plasma clearance of LPS and hepatic LPS uptake. (A) LPS levels in plasma were measured by microplate reader 5 min, 1 and 6 hours post injection of a non-lethal dose of FITC-conjugated E.coli LPS (5 mg/kg) into tail vein of control (wild type) and Ldlr-/- mice. (B) Data are presented as mean fluorescence intensity of homogenized liver tissue detected by the microplate reader and normalized to mice injected with non-fluorescent LPS and tissue weight, mean±SEM, (n = 5). Analyzed by two-way ANOVA.

To test the role of LDLR in LPS clearance from plasma, FITC-LPS (5 mg/kg) was injected intravenously into wild type and *Ldlr-/-* mice and LPS fluorescence in plasma was measured 5 minutes, 1 hour and 6 hours post injection. After 5 minutes the level of fluorescence was the same in both groups indicating that both groups received the same dose of FITC-conjugated LPS (Fig 2A). LPS fluorescence in plasma of wild type mice was significantly (p<0.0001) reduced after 1 hour and dropped even further by 6 hours (p<0.001) indicating clearance from plasma. In *Ldlr*-/- mice LPS clearance from plasma was decreased compared to wild type mice after 1 (p = 0.0021) and 6 hours (p = 0.0249) (Fig 2A) suggesting that *in vivo* clearance of LPS from plasma is LDLR-dependent.

### Murine Ldlr knockout decreases hepatic LPS uptake

We next investigated whether the reduced plasma clearance of LPS was correlated with reduced hepatic uptake of LPS in *Ldlr*-/- mice. Hepatic LPS fluorescent intensity in wild type mice was significantly higher at 6 hours compared to 1 hour (p = 0.005), which correlated with greater LPS plasma clearance at this time point (Fig 2B). Hepatic LPS uptake by *Ldlr*-/- mice was significantly decreased compared to wild type mice both 1 (p = 0.0044) and 6 hours (p<0.0001) post LPS injection. Hepatocytes represent the major cell type in the liver, thus we believe that vast LPS fluorescence in the liver is due to uptake by hepatocytes. However we cannot rule out the effect of Kuppfer cells, which requires further investigation. These *in vivo* data indicate that hepatic LPS clearance is, at least in part, via an LDLR-dependent mechanism.

### *In vitro* confirmation of LPS uptake via LDLR in murine hepatocytes

**Fig 3:**
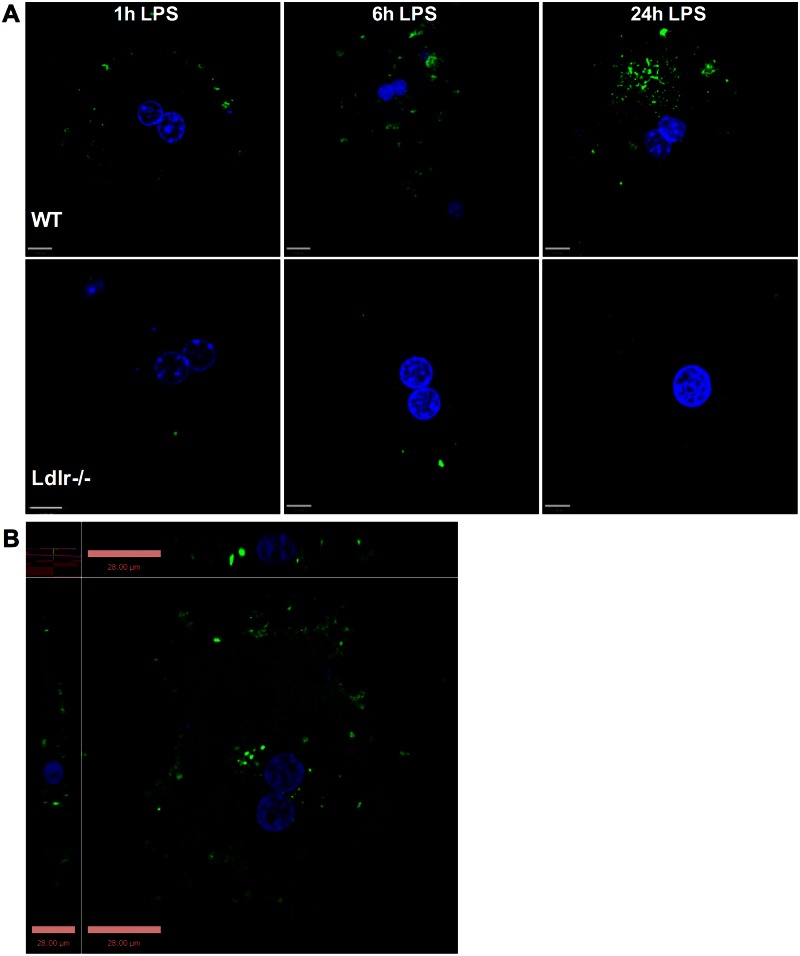
LPS uptake into hepatocytes is LDLR-dependent. (A) Primary hepatocytes were isolated from wild type and Ldlr-/- mice, plated on 12 mm coverslips precoated with Collagen I in DMEM medium with 20% Bovine Serum, 100 units/mL penicillin, and 100 units/mL streptomycin. Cells were allowed to attach to coverslips for 6–12 hours prior the treatment with 2.5 ug/mL Alexa Fluor 488–conjugated LPS was added for 1, 6 and 24 hours total. Coverslips were fixed with 4% paraformaldehyde for 20 min and stained with DAPI to identify nuclei (blue). Images are representative of three independent experiments. (B) 2D image of primary hepatocytes isolated form WT mice and treated with 2.5 μg/mL Alexa Fluor 488–conjugated LPS for 24 hours. LPS uptake was visualized using Leica Inverted Fluorescence microscope (x63 magnification) and analyzed using VOLOCITY software (PerkinElmer Inc.).

Whole-tissue *in vitro* measurements do not distinguish between cell types found in the liver (e.g. hepatocytes, Kuppfer cells, etc). To address this issue we used primary hepatocytes isolated from wild type and *Ldlr*-/- mice and imaged fluorescent LPS uptake and internalization in the presence of serum (Fig 3B). Primary murine hepatocytes show significant LPS uptake at the cell periphery at 1 hour and further uptake and internalization of LPS towards the nucleus area by 6 and 24 hours (Fig 3A, top panel). LPS uptake was greatly reduced in primary murine hepatocytes from *Ldlr*-/- mice at all time points indicating a significant role for the LDLR in LPS uptake by hepatocytes (Fig 3A, bottom panel).

### LPS uptake is mediated by the LDLR in human hepatocytes

**Fig 4:**
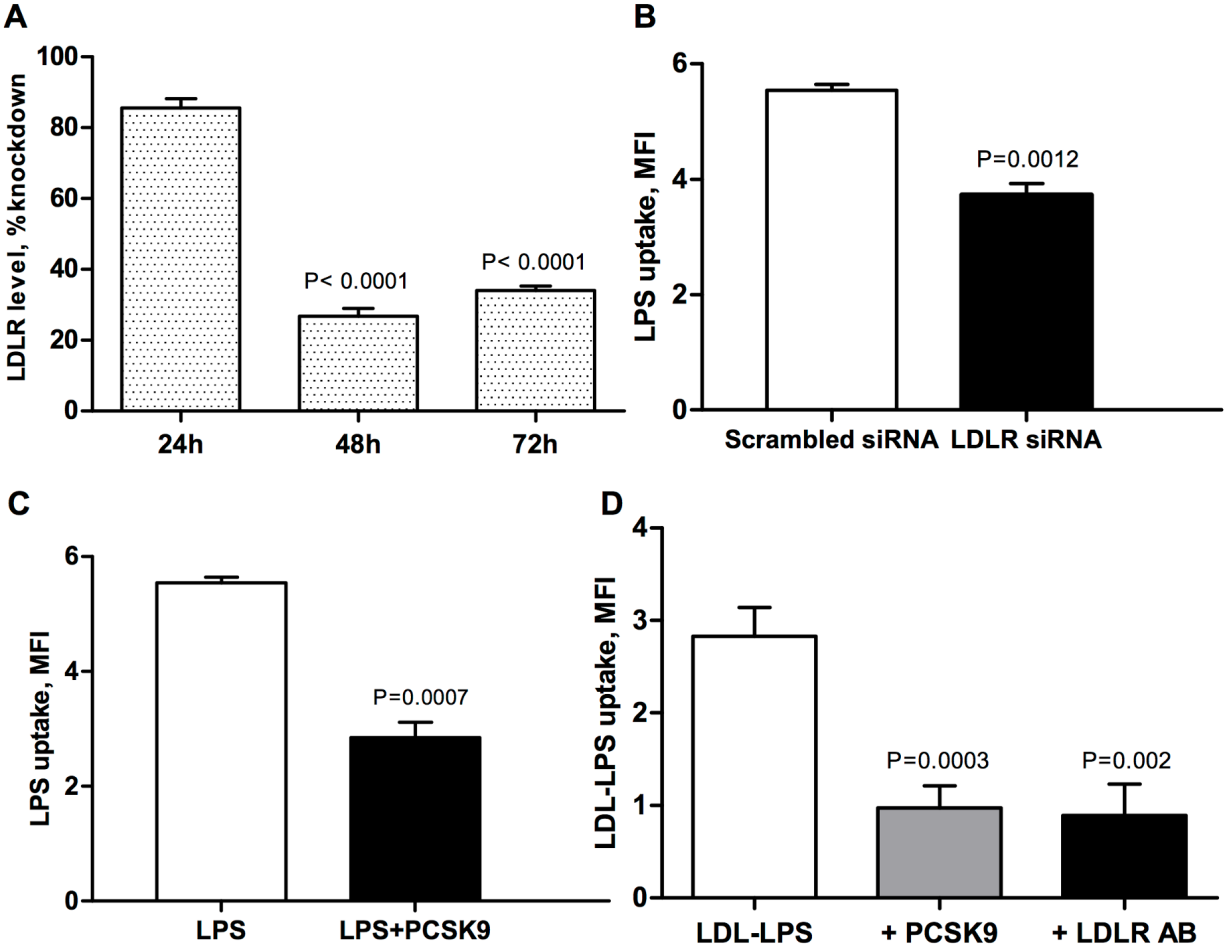
HepG2 cells clear LPS in LDLR-dependent manner. (A) The LDLR specific and scrambled siRNAs were reversely transfected into HepG2 cells. After 24, 48 and 72 hrs, the cells were detached and stained with LDLR-PE conjugated antibody. The LDLR levels were analyzed by flow cytometry. Data presented as percent of mean fluorescence intensity from scrambled control, mean±SEM (n = 3). Highest knockdown efficiency (73%) was achieved 48 hours after transfection. (B) Effect of LDL receptor knockdown on LPS uptake. HepG2 cells were reversely transfected with siRNA targeting LDLR and control scrambled siRNA. Cells were treated with Alexa Fluor 488-conjugated LPS (2.5 μg/ml) 48 hours after transfection for 24 hours. LPS uptake was measured by flow cytometry. Data presented as mean fluorescence intensity, mean±SEM (n = 3). (C) PCSK9 incubation decreased LPS uptake by HepG2 cells. Scrambled siRNA was reversely transfected into HepG2 cells, than treated similarly as described above with PCSK9 as a complementary approach to decreasing LDLR. Data presented as mean fluorescence intensity, mean±SEM (n = 3). (D) HepG2 cells uptake LDL-labeled LPS complex via LDLR. Freshly isolated LDL form healthy donors was labeled with Alexa-488 Fluor LPS, and separated with the Sephadex column. HepG2 cells were pretreated with PCSK9 (3 μg/mL) or LDLR AB (3 μg/mL) to block the LDLR levels before adding LDL-LPS complex (50 μg/mL). The LDL-LPS uptake was analyzed by flow cytometry. Data presented as mean fluorescence intensity, mean±SEM (n = 3).

To confirm that LDLR is involved in LPS uptake by human hepatocytes *in vitro*, we used siRNA to knockdown the LDLR (Fig 4A and 4B) in human hepatic cell line, HepG2. LPS uptake was significantly decreased at 24 hours in cells after siRNA knockdown of the LDLR compared to cells treated with scrambled siRNA (p = 0.0012) (Fig 4B). We then used PCSK9 treatment as a complementary approach for decreasing LDLR. We found that PCSK9 incubation also significantly decreased LPS uptake by HepG2 cells (p = 0.0007) (Fig 4B and 4C). Finally, to fully demonstrate that LDLR mediates LPS uptake, HepG2 cells were treated with LDL-LPS complex. LDL was obtained by fresh plasma isolation from healthy donors and ultracentrifugation separation of lipoproteins (S1 Fig). Isolated LDL was then labeled with Alexa-488 Fluor LPS (Fig 4D). We used an LDLR antibody and PCSK9 to decrease LDLR levels. LDL-LPS complex uptake was significantly down-regulated by pretreatment with PCSK9 (p = 0.0003) or LDLR antibody (p = 0.002) (Fig 4D), suggesting that LPS can be bound to LDL and taken up by the LDL receptor.

### Potential role of other PCSK9-sensitive receptors

**Fig 5:**
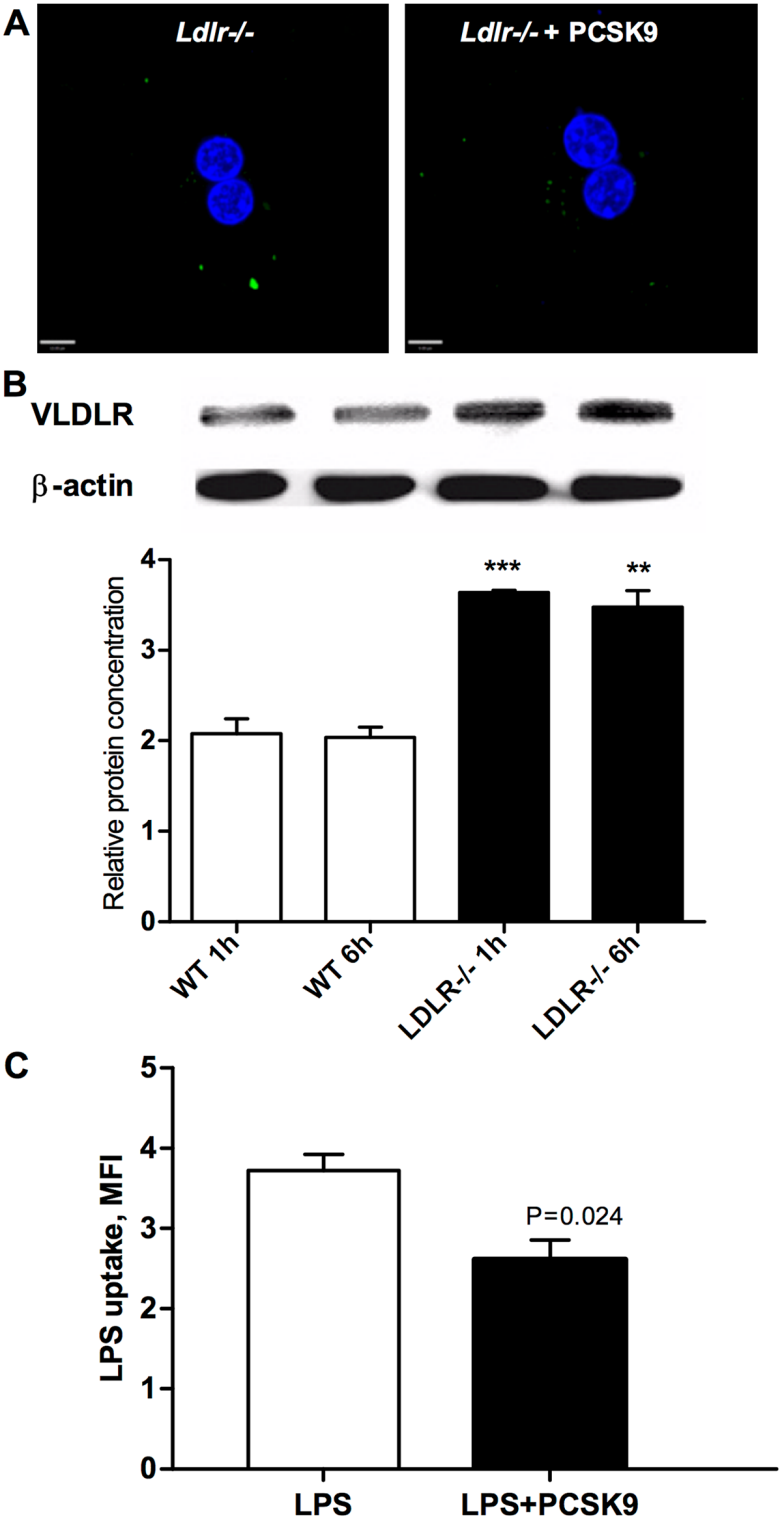
LPS uptake is mediated by the LDLR in human hepatocytes. (A) PCSK9 further reduced LPS uptake in Ldlr-/- cells. Primary hepatocytes were isolated from Ldlr-/- mice. Cells were allowed to attach to coverslips for 6–12 hours than treated with 2.5 ug/mL Alexa Fluor 488–conjugated LPS for 6 hours total. Coverslips were fixed with 4% paraformaldehyde for 20 min and stained with DAPI to identify nuclei (blue). LPS uptake was visualized using Leica Inverted Fluorescence microscope (x63 magnification) and analyzed with VELOCITY software. Images are representative of three independent experiments. (B) Ldlr-/- mice have increased expression of VLDLR in a liver. VLDR receptor protein expression by Western blot in wild type and Ldlr-/- mouse liver at time points 1 hour and 6 hours after injection of LPS. Data presented as mean±SEM, (***P = 0.0007, compared with control 1h; **P = 0.0026, compared with control 6h, n = 3). (C) PCSK9 further altered uptake of LPS in HepG2 cells with LDLR knockdown. HepG2 cells were reversely transfected with siRNA targeting LDLR and control scrambled siRNA. Recombinant human PCSK9 (3 μg/ml) was added 4 hours before and 4 and 19 hours after LPS treatment. Cells were treated with Alexa Fluor 488-conjugated LPS (2.5 μg/ml) 48 hours after transfection for 24 hours. LPS uptake was measured by flow cytometry. Data presented as mean fluorescence intensity, mean±SEM (n = 3).

As shown above (Fig 2B) we observe hepatic uptake of LPS in *Ldlr*-/- mice despite the absence of LDLR in the liver of these mice. Thus there must be alternative mechanism(s) involved in LPS uptake by liver of the mice lacking LDLR. Interestingly PCSK9 further reduced LPS uptake in *Ldlr*-/- cells (Fig 5A) suggesting that PCSK9 might also act on another LDLR-independent mechanism of LPS uptake. PCSK9 can also promote degradation of the VLDL receptor (VLDLR) [14]. VLDLR, which is usually not expressed in the liver of humans, shows ectopic upregulation in hepatocytes of *Ldlr*-/- mice [15]. We confirmed that VLDLR is expressed in liver of *Ldlr-/-* mice to a greater extent than in wild type mice by Western blot analysis of liver (Fig 5B) thus providing a possible explanation for the effect of PCSK9 on hepatic LPS uptake in *Ldlr*-/- mice. Interestingly, following LDLR siRNA knockdown in HepG2 cells, treatment with recombinant PCSK9 further decreased uptake of LPS (p = 0.024) (Fig 5C). Thus, a non-LDLR but PCSK9-regulated mechanism for LPS clearance may also be important in humans.

## Discussion

While decreased PCSK9 function improves clinical outcomes in murine and human sepsis [3], the mechanisms involved have not been fully elucidated. Hepatocytes play an important role in LPS clearance [16, 17]. Therefore, PCSK9-sensitive lipoprotein receptors might be involved in hepatocyte-mediated LPS clearance [3] but this has not been shown directly. Accordingly we tested the hypothesis that LPS is cleared from the circulation via hepatocyte LDL receptors.

We found that hepatocyte LDLR are involved in clearance of circulating LPS. Clearance of i.v. injected LPS was reduced substantially in *Ldlr*-/- mice compared to wild type controls and, simultaneously, hepatic uptake of LPS was also reduced in *Ldlr*-/- mice. These observations confirm that the liver plays a major role in LPS clearance. The liver consists of multiple cell types and therefore we could not pinpoint involvement of a specific cell type based on these data alone. For example, Kupffer cells (KC) have been implicated in LPS clearance via TLR4 [17]. It has been postulated that within the blood, a complex of LPS and the LPS-binding protein (LBP) shows an affinity for CD14 on macrophages such as Kupffer cells [18] leading via cell surface receptors to produce inflammatory cytokines, including interleukin-1 (IL-1) [19], interleukin-10 (IL-10) [19] and tumor necrosis factor-alpha (TNF-α) [19] that activate hepatocytes to express pro-inflammatory molecules [12]. Evidence suggests that hepatocytes play an important role in inflammation during sepsis [20]. Little is known about the mechanisms involved in the exact mechanisms of hepatic uptake of pathogen lipids.

Previously several receptors have been reported to be activated in response to LPS, including scavenger receptors in Kupffer cells [21], TLR4 [8, 22] and TLR2 [23] in hepatocytes, and possibly other routes [24]. However we clearly demonstrated that LPS is cleared from circulation predominantly by LDLR. LPS administration increases VLDL and LDL levels while decreasing HDL levels [25]. In serum, LPS predominantly binds to very low density lipoprotein (VLDL) and LDL, which results in the inactivation of LPS [7, 26–28]. Therefore LPS sequestered within any lipoprotein particles cannot bind TLRs and alternative route (e.g LDLR) should exist to explain hepatic LPS uptake [29].

To specifically investigate the role of LDLR-dependent mechanism in hepatocytes we therefore examined LPS clearance by hepatocytes *ex vivo*. We found that primary hepatocytes from *Ldlr*-/- mice had greatly decreased LPS uptake. These experiments were done in the presence of serum in order to provide lipoproteins and transfer proteins for LPS carriage [6]. Interestingly, we found that LPS is rapidly taken up by primary hepatocytes into the cell periphery. Over the course of 6 hours LPS is internalized and transported towards the hepatocyte nucleus area.

Since murine hepatocytes, and specifically those from genetically modified mice, may function somewhat differently from human hepatocytes, we tested for replication of these finding in a human hepatocyte cell line using analogous but different strategies. In the HepG2 immortalized human hepatocyte cell line, *LDLR* silencing similarly resulted in decreased LPS uptake. PCSK9 treatment reduces LDLR density on hepatocytes and, therefore, was another independent strategy to test our hypothesis. Incubation with PCSK9 indeed reduced LPS uptake by hepatocytes. Toxic pathogen lipids such as LPS are carried in lipoprotein particles including HDL, LDL, and VLDL [7, 27] which results in the inactivation of inflammatory effect of LPS by sequestration [7, 27, 30–32]. For our *in vitro* studies, the incubation medium contained 20% serum. To specifically emphasize the LDL-LPS binding, we have shown that HepG2 cells rapidly uptake LDL-LPS complex, while LDLR blocking antibody, as well as PCSK9 significantly reduced LDL-LPS uptake, but not HDL-LPS (data not shown). Taken together, these observations directly demonstrate that LPS can be bound to LDL and cleared from the circulation by hepatocytes via the LDLR.

PCSK9, a key regulator of LDLR hepatic activity, binds the LDLR and promotes its internalization and degradation in lysosomal compartments in hepatocytes [33]. Decreased lipoprotein levels are observed in sepsis, for example HDL levels drop by 40–70% in septic patients [31]. These decreased levels upregulate PCSK9 transcription so that PCSK9 levels are increased during human sepsis [32]. Elevated PCSK9, in turn, decreases LDLR density on hepatocytes. This is consistent with the observation that LPS administration decreases LDL receptor protein levels in the liver [34, 35]. Thus, PCSK9 may be an important regulator of hepatic LDLR–dependent uptake of LPS during sepsis.

It is important to note that acute infection/inflammation has been shown to affect LDL clearance from the circulation in a species-specific manner. In rats, LPS significantly inhibits the clearance of LDL from the circulation by posttranscriptional down-regulation of LDLR during inflammation; whereas in human HepG2 cells, IL-1 and TNF increase LDL receptor activity [25]. The differences may explain the species-specific response in cholesterol metabolism commonly seen during the acute inflammatory response. These discrepancies in LDL clearance during acute infection may be explained by differences in blood lipoprotein profiles between rodents and primates. Lipoproteins in mice are dominated by HDL rather than LDL compared to humans; mice express lipopolysaccharide binding protein (LBP) and phospholipid transfer protein (PLTP), but lack cholesterol ester transfer protein (CETP) [36]. CETP has a low affinity for LPS [37] but may have an indirect effect by altering the lipoprotein profile in blood. Thus future studies using “humanized” mice developed with the specific goal of emulating a human lipoprotein profile may help to answer these questions, e.g. CETP-ApoB100 mice, which carry both human CETP and human ApoB100 transgenes. Whether PCSK9 plays a role in these species-specific differences is unknown.

Other receptors and members of the LDLR family might also play a role. For example, PCSK9 enhances degradation of the closest LDLR family member, VLDLR [38], and regulates VLDLR protein levels in adipose tissue [39]. We found that VLDLR protein, usually not expressed or expressed at very low levels in the liver of wild type mice [40], is upregulated in *Ldlr*-/- mice. Thus, ectopic expression of VLDLR could partially explain the effect of PCSK9 on hepatic LPS uptake in *Ldlr*-/- mice. Other receptors, including scavenger receptors in Kupffer cells [41], TLR4 [42] and TLR2 [42] in hepatocytes, and possibly other routes [19] could conceivably be involved in hepatic clearance of LPS. Yet the majority of the effect in any of our experimental models appeared to be explained by the LDLR.

Increasingly severe hepatic dysfunction (acute or chronic) dramatically increases risk of death in patients who have sepsis [43]. Our prior studies [3] and the current confirmation of the critical role of hepatic PSCK9-regulated LDLR expression in LPS clearance suggest a novel understanding of why hepatic dysfunction is such an important risk in sepsis.

In summary, our results demonstrate that human and murine hepatocytes clear LPS from the circulation via the LDLR. Furthermore, PCSK9 regulates LPS clearance from the circulation during sepsis by downregulation of hepatocyte LDLR. These findings might elucidate an entirely novel simple clinical approach–i.e. administration of PCSK9 inhibitors—to improve adverse outcomes due to inflammation triggered by pathogen toxins in patients with sepsis. Further clinical implications are the role of underlying hepatic dysfunction in sepsis by understanding the mechanism of pathogen lipid clearance.

## Supporting Information

S1 Fig LPS bounds to human LDL. LDL isolated from healthy volunteers was labeled with Alexa 488 conjugated LPS. The protein concentration graph shows the maximum at fraction 4 and 5 and the minimum at rest of the fractions. This indicates the presence of LDL in fraction 4 and 5. The fluorescence level graph shows the 2 peaks: one at fraction 4 and 5 and another at fraction 9 and 10. The left peak is fluorescence from LDL-bound LPS and the right peak is fluorescence from free LPS. The labeling of LDL with LPS was confirmed by overlapping the graphs of protein concentration and fluorescence level. For further experiments, the fraction 4 and 5 were pooled and the protein concentration and fluorescence level were measured by BCA assay and fluorescence plate reader, respectively.
(TIFF)
