# Correction: Lipopolysaccharide Is Cleared from the Circulation by Hepatocytes via the Low Density Lipoprotein Receptor

**DOI:** 10.1371/journal.pone.0160326

**Published:** 2016-07-26

**Authors:** Elena Topchiy, Mihai Cirstea, HyeJin Julia Kong, John H. Boyd, Yingjin Wang, James A. Russell, Keith R. Walley

References 20 and 42 are cited in error in the published article. References 20 and 42 should be removed from the article. Reference 20 is: Abazov VM, Abbott B, Abolins M, Acharya BS, Adams M, Adams T, et al. Search for associated higgs boson production WH—>WWW*—>l+/-nul'+/-nu'+X in pp collisions at square root s = 1.96 Te V. Physical review letters. 2006;97(15):151804. Epub 2006/12/13. pmid:17155320. Reference 42 is: Abazov VM, Abbott B, Abolins M, Acharya BS, Adams M, Adams T, et al. Measurement of the Bs(0) lifetime using semileptonic decays. Physical review letters. 2006;97(24):241801. Epub 2007/02/07. pmid:17280267.
