# Supplementary figures and images for: Lipopolysaccharide Is Cleared from the Circulation by Hepatocytes via the Low Density Lipoprotein Receptor
Source: PLoS One. 2016 May 12;11(5):e0155030. doi: 10.1371/journal.pone.0155030 (PMC4865154; doi:10.1371/journal.pone.0155030)

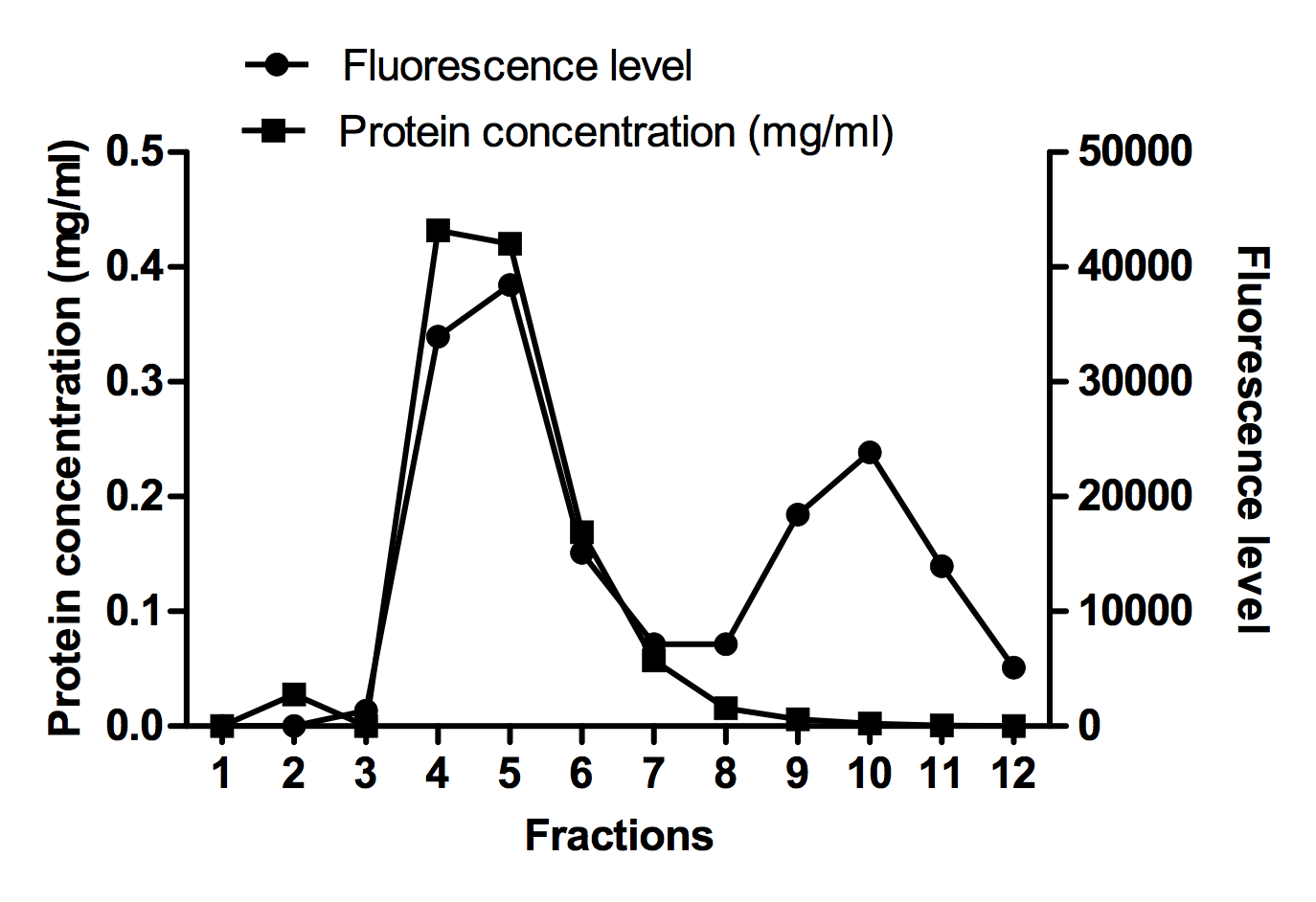

Supplement: S1 Fig — LDL isolated from healthy volunteers was labeled with Alexa 488 conjugated LPS. The protein concentration graph shows the maximum at fraction 4 and 5 and the minimum at rest of the fractions. This indicates the presence of LDL in fraction 4 and 5. The fluorescence level graph shows the 2 peaks: one at fraction 4 and 5 and another at fraction 9 and 10. The left peak is fluorescence from LDL-bound LPS and the right peak is fluorescence from free LPS. The labeling of LDL with LPS was confirmed by overlapping the graphs of protein concentration and fluorescence level. For further experiments, the fraction 4 and 5 were pooled and the protein concentration and fluorescence level were measured by BCA assay and fluorescence plate reader, respectively. (TIFF) [file pone.0155030.s001.tiff]
